# Targeted next‐generation sequencing of 22 mismatch repair genes identifies Lynch syndrome families

**DOI:** 10.1002/cam4.628

**Published:** 2016-01-25

**Authors:** Bente A. Talseth‐Palmer, Denis C. Bauer, Wenche Sjursen, Tiffany J. Evans, Mary McPhillips, Anthony Proietto, Geoffrey Otton, Allan D. Spigelman, Rodney J. Scott

**Affiliations:** ^1^ School of Biomedical Sciences and PharmacyFaculty of Health and MedicineUniversity of NewcastleNewcastleNew South WalesAustralia; ^2^ Centre for Information‐Based MedicineHunter Medical Research InstituteNewcastleNew South WalesAustralia; ^3^ CSIRO Digital ProductivitySydneyNew South WalesAustralia; ^4^ Department of Laboratory Medicine Children's and Women's HealthNorwegian University of Science and TechnologyTrondheimNorway; ^5^ Department of Pathology and Medical GeneticsSt Olavs University HospitalTrondheimNorway; ^6^ Hunter Area Pathology ServicePathology NorthHunter New England Area HealthNewcastleNew South WalesAustralia; ^7^ Hunter Centre for Gynaecological CancerHunter New England Area HealthNewcastleNew South WalesAustralia; ^8^ School of Medicine and Public HealthFaculty of Health and MedicineUniversity of NewcastleNew South WalesAustralia; ^9^ Hunter Family Cancer ServiceHunter New England Area HealthNewcastleNew South WalesAustralia; ^10^ St Vincent's Hospital Clinical SchoolUniversity of NSW and Hospital Cancer Genetics ClinicThe Kinghorn Cancer CentreSydneyNew South WalesAustralia

**Keywords:** Cancer genetics, colorectal cancer, inherited cancer

## Abstract

Causative germline mutations in mismatch repair (MMR) genes can only be identified in ~50% of families with a clinical diagnosis of the inherited colorectal cancer (CRC) syndrome hereditary nonpolyposis colorectal cancer (HNPCC)/Lynch syndrome (LS). Identification of these patients are critical as they are at substantially increased risk of developing multiple primary tumors, mainly colorectal and endometrial cancer (EC), occurring at a young age. This demonstrates the need to develop new and/or more thorough mutation detection approaches. Next‐generation sequencing (NGS) was used to screen 22 genes involved in the DNA MMR pathway in constitutional DNA from 14 HNPCC and 12 sporadic EC patients, plus 2 positive controls. Several softwares were used for analysis and functional annotation. We identified 5 exonic indel variants, 42 exonic nonsynonymous single‐nucleotide variants (SNVs) and 1 intronic variant of significance. Three of these variants were class 5 (pathogenic) or class 4 (likely pathogenic), 5 were class 3 (uncertain clinical relevance) and 40 were classified as variants of unknown clinical significance. In conclusion, we have identified two LS families from the sporadic EC patients, one without a family history of cancer, supporting the notion for universal MMR screening of EC patients. In addition, we have detected three novel class 3 variants in EC cases. We have, in addition discovered a polygenic interaction which is the most likely cause of cancer development in a HNPCC patient that could explain previous inconsistent results reported on an intronic *EXO1* variant.

## Introduction

Surveillance programs for patients with an inherited predisposition to colorectal cancer have proven efficacy in the reduction of morbidity and mortality by up to 65% 1. Colorectal cancer (CRC) is a heterogeneous disease and one of the most common cancers worldwide 2. CRC can be categorized into two groups; one associated with chromosomal instability and the other with microsatellite instability (MSI) 3. An inherited form of the latter, called hereditary nonpolyposis colorectal cancer (HNPCC)/Lynch syndrome (LS), is associated with the inactivation of genes involved in DNA mismatch repair (MMR). MMR deficiency has been observed in 15–17% of all primary CRC 4, 5, 30% of endometrial cancer (EC) 6, and approximately 10% of ovarian tumors 7.

The identification of germline mutations in families with LS accounts for only ~50% of all families that fulfil the Amsterdam criteria 8. Patients with germline DNA MMR mutations in *MLH1, MSH2, MSH6* and *PMS2* or mutations in *EPCAM* (leading to impaired DNA repair through epigenetic silencing of MSH2) are defined as having LS 9, 10, 11, whereas the mutation negative patients are referred to as belonging to the entity known as HNPCC and only have a clinical diagnosis of the disease according to the Amsterdam criteria. On top of the high risk of CRC and EC, patients are also at greater risk of developing other epithelial malignancies 12, 13, 14. The primary function of MMR genes is to eliminate base‐base mismatches and insertion‐deletion loops which arise as a consequence of DNA polymerase slippage during DNA replication 15. MMR confers several genetic stabilization functions: It corrects DNA biosynthesis errors, ensures the fidelity of genetic recombination and participates in the earliest steps of checkpoint and apoptotic responses 16, 17. The absence of an effective MMR pathway is presumed to lead to the accumulation of mutations and an increased risk of disease.

EC is the most common gynecological malignancy in Western countries 18, yet the genetic basis of the disease is poorly understood. The disease has a strong association with obesete, the higher the BMI the higher the risk of EC 19. MSI has been reported in 22–45% of sporadic EC 20, 21, 22. The rate of MSI tumors reported in EC is much higher compared to other cancers, illustrating that abnormalities in the DNA MMR pathway appear to play a central role in EC development 23. But MSI analysis, together with the clinical diagnostic criteria, is thought to have limited value in screening for LS‐associated EC as only a small portion of patients with MSI tumors have mutations in MMR genes and about 15% of EC cases that did not fulfil the clinical diagnostic criteria of the disease had MMR gene mutations^24^. Two of the largest studies to date have found that 1.8–2.1% of EC cases have LS 25, 26. Taken together with the fact that only half of patients with a clinical diagnosis of HNPCC have mutations in MMR genes, this shows that our efforts should be focused on the development of new and/or more thorough mutation detection approaches.

In this study we used targeted next‐generation sequencing (NGS) to examine 22 genes involved in the DNA MMR pathway in constitutional DNA from 14 HNPCC patients and 12 EC patients, plus 2 positive controls and a replicate.

## Materials and Methods

The study complies with the requirements of the Hunter New England Human Research Ethics Committee and the University of Newcastle Human Research Ethics Committee, Newcastle, NSW, Australia. Written informed consent was obtained from all participants.

### Participants

HNPCC probands referred to Hunter Area Pathology Service (HAPS, Pathology North) for genetic testing between the years 1997 and 2010 were used in this study. Fourteen unrelated HNPCC probands screened for mutations in *MLH1*,* MSH2*,* MSH6* and/or *PMS2* using a combination of DNA sequencing and multiplex ligation‐dependent probe amplification (MLPA) assays who were found to be mutation negative where included in the study. All patients had a diagnosis of CRC and conformed to the Amsterdam II criteria. Immunohistochemistry (IHC) and/or MSI results indicated a loss of expression of one or more of the four MMR genes (*MLH1, MSH2, MSH6* and *PMS2*) where available for seven of these patients. Two mutation‐positive samples and a replicate were included as internal controls.

DNA was also included from patients that were confirmed histologically as EC patients derived from the Hunter Centre for Gynaecological Cancer, John Hunter Hospital between the years of 2005 and 2006. A total of 12 EC patients were included in the study which comprised six patients with an additional diagnosis of CRC, three patients with colorectal adenomas, one patient with a renal cancer, one with breast cancer and one with a family history of CRC.

### Target next‐generation sequencing

Constitutional DNA extracted from whole blood from the 29 cases (14 HNPCC and 12 EC cases, plus two LS cases as positive controls (one as replicate) were sent to BGI, China for targeted NGS. The target region (exons and introns) of 22 MMR genes; *MLH1*,* MSH2*,* MSH6*,* PMS2*,* MSH3*,* PMS1*,* MLH3*,* EXO1*,* RFC1*,* RFC2*,* RFC3*,* RFC4*,* RFC5*,* PCNA*,* LIG1*,* RPA1*,* RPA2*,* RPA3*,* POLD1*,* POLD2*,* POLD3* and *POLD4,* with a total size of 1.161 Mb were screened for causative germline mutations. BGI conducted the sequencing according to NimbleGen human custom array (Roche NimbleGen, Madison, Winsconsin, USA) and Illumina (HiSeq2000, San Diego, California, USA) protocols. Sequencing for each captured library was performed independently to ensure 100× coverage. Raw image files was processed by Illumina base calling Software 1.7 with default parameters and the sequences of each individual were generated as 90 bp paired‐end reads. The NimbleGen chip coverage was 87.8% of the target region (1,066,805 bp of 1,215,301 bp covered), with a maximum mismatch of one (mm1) in every probe.

### Bioinformatics

NGSANE 27 v0.4.0.2 was used to process the raw sequence files. Briefly, the pipeline maps the fastq files using burrows‐wheeler aligner (BWA) 28 to the human reference genome (b37, as obtained from GATK reference bundle), with subsequent score recalibration and realignment, using GATK 29 v2.8‐1 with dbSNP v135 as guide. Variants were called over all alignment files simultaneously using GATK and dbSNP, HapMap v3.3 and the 1000 genomes project variant information was used as resource for the variant recalibration. Larger structural variants were called with Pindel V0.2.5 30. Quality control and reporting was done using NGSANE.

### Annotation of genetic variants

A script within the ANNOVAR package 31, TABLE_ANNOVAR, was used with the following annotations requested: refGene (gene name); cytoBand (region); SIFT score, PolyPhen score, Phylop score, LRT score and GERP++ score (conservation score) – all annotations for nonsynonymous variants; 1000 genomes project (2012 release); dbsnp138; and Clinvar_20140303 (database that archives and aggregates information about relationship among variation and human health). The following categorical predictions for nonsynonymous variants were applied: SIFT score; deleterious (≤0.05) and tolerated (>0.05), PolyPhen 2 HDIV; probably damaging (≥0.957), possibly damaging (>0.453 and <0.956) and benign (<0.452), PolyPhen 2 HVar; probably damaging (≥0.909), possibly damaging (>0.447 and <0.909) and benign (<0.446), LRT; deleterious (>0.958); GERP++ and Phylop scores (>2.0 considered potentially pathogenic). Single‐nucleotide variants (SNVs) were also investigated using Alamut Visual (Interactive Biosoftware, Rouen, France) to aid in the interpretation of the pathogenic status of the variant. The International Society of Gastrointestinal Hereditary Tumours (InSIGHT) classification system 32 will be used to classify; class 5 (pathogenic), class 4 (likely pathogenic) and class 3 (uncertain) variants. Literature searches, predication softwares (i.e., Alamut) and a frequency of the variant of >1% will be used to classify variants of unknown clinical significance.

## Results

The demographic characteristics of HNPCC and EC patients in the current study can be seen in Tables 1 and  2, respectively. The average age of diagnosis of CRC in the HNPCC cases were 48 years, while the average age of diagnosis of EC in the EC cases were 62 years of age. Six of the EC cases were obese, three overweight, one was normal and two were of unknown BMI status, see Table 2. All the HNPCC cases fulfilled the Amsterdam II criteria, while family history of cancer was observed in 7/12 EC patients.

**Table 1:** Sample demographics – HNPCC cases

Sample ID	Disease	Age of diagnosis (CRC)	Other cancer diagnosis	IHC results (if known)	Genes screened previously	Polymorphisms detected during previous mutation screening
1	HNPCC	63			MLH1/MSH2/MSH6	
2	HNPCC	45			MLH1/MSH2/MSH6	
3	HNPCC	42	Breast cancer	−ve MSH2/MSH6	MSH2/MSH6	
6	HNPCC	63		−ve MLH1	MLH1/MSH2	MLH1, ex18, c.2101C>A, p.Gln701Lys^a^
8	HNPCC	50s			MLH1/MSH2/MSH6/PMS2	
9	HNPCC	39		−ve MLH1	MLH1/MSH2/PMS2	
10	HNPCC	21		−ve MLH1	MLH1	MLH1, ex8, c.655A>G, p.Ile219Val^a^
11/31	LS (internal control)	37		No tissue available	MLH1/MSH2/MSH6/PMS2	MSH2, ex6, c.965G>A, p.Gly322Asp^a^ (11 + 31) MSH6, ex2, c.431G>T, p.Ser144Ile^a^ (11 + 31) PMS2, ex10, c.989‐296_1144 + 706del, p.Glu330_Glu381del (Class 5)
12	HNPCC	45		−ve MSH6	MSH6	
13	HNPCC	51		−ve MLH1/PMS2	MLH1/MSH2	
14	HNPCC	21			MLH1/MSH2	
17	LS (control)	51	EC			
18	HNPCC	68		−ve MLH1	MLH1	MLH1, ex8, c.655A>G, p.Ile219Val^a^
32	HNPCC	66			MLH1/MSH2/MSH6/MUH/MUTYH	
33	HNPCC	64			MLH1/MSH2/PMS2	
35	HNPCC	48			MLH1/MSH2	MLH1, ex8, c.655A>G, p.Ile219Val^a^

a Detected with GATK in this study.

**Table 2:** Sample demographics – endometrial cancer (EC) cases

Sample ID	Disease	Age of diagnosis	Other cancer diagnosis	Family history of cancer	BMI (classification)
19	EC	62	CRC	Yes	Unknown
20	EC	60	CRC	No	31.2 (obese)
21	EC	57	Adenocarcinoma	Yes	45.7 (obese)
22	EC	76	CRC, skin cancer, ovarian cancer	Yes	38.5 (obese)
23	EC	71	CRC, ovarian cancer	Yes	32.5 (obese)
24	EC	65	CRC	No	Unknown
25	EC	63	Bowel polyps, skin cancer	Yes	29.4 (overweight)
26	EC	55	CRC	No	29.0 (overweight)
27	EC	53	Bowel polyps	No	40.4 (obese)
28	EC	44	Family history of CRC	Yes	22.0 (normal)
29	EC	62	Renal cancer	No	37.5 (obese)
30	EC	79	Breast cancer	Yes	26.9 (overweight)

For all samples, at least 97.2% of reads mapped to the reference genome. All samples generated a mean reading depth (coverage) of at least 110×. In the 29 samples we detected; 897 indels (insertion/deletions) and 3900 SNVs using GATK, in addition we detected 3830 structural variants using Pindel. From the annotation we identified 5 exonic variants, 42 nonsynonymous SNVs and one intronic variant of significance. The variants of significance are listed in three tables according to variant classification; deleterious or probably deleterious variants – class 5/4 (Table 3), variants of uncertain clinical relevance – class 3 (Table 4) and probably benign variants or polymorphisms – variants of unknown clinical significance (Table 5).

**Table 3:** Deleterious or probably deleterious variants detected in the sample cohort (class 5 – pathogenic and class 4 – likely pathogenic variants)

Geneexon	Ref. sequence	Variant	Found in no. of cases (sample ID)	Comments	ANNOVAR annotation
MLH1Exon 1	NM_000249.2	c.116G>A p.Cys39Tyr	*n* = 1 (#21 = EC)	Single nucleotide variant (SNV) LOVD: MLH1_00026, class 4 likely pathogenic (last nucleotide in exon)	SIFT: deleterious (0.02), PolyPhen 2 HDIV/HVAR: probably damaging/potentially pathogenic, Gerp++: deleterious (5.68), 1000 g2012: Novel, Clinvar: probable pathogenic (Lynch)
MSH6Exon 3	NM_000179.2	c.458_627del p.Ser154Argfs*8	*n* = 1 (#20 = EC)	Frameshift deletion LOVD: MSH6_00336, class 5 pathogenic	
MSH2Exon 1	NM_000251.2	c.186_187dup p.Val63Glyfs*2	*n* = 1 (#17 = LS)	Positive control sample, Frameshift insertion 2 bp insertion that creates a frame shift that ends in a STOP codon 1 position downstream LOVD: MSH2_00762, class 5 Pathogenic	

**Table 4:** Variants of uncertain clinical relevance detected in the sample cohort (class 3 variants)

GeneExon	Ref. sequence	Variant (SNV)	Found in no. of cases (sample ID)	Rs number	MAF	Comments
EXO1Intron 14	NM_003686.4	c.2212‐1G>C	*n* = 1 (#18 = HNPCC)	rs4150000	0.0014	Splice site substitution located in the acceptor splice site in intron 14. The consequence of this change is not predictable, but a skip of exon 15 is very likely.Gerp++ score (conservation score): Deleterious with 4.94
LIG1Exon 11	NM_001289064.1	c.980C>T p.Pro327Leu	*n* = 1 (#24 = EC)	No rs number	No frequency data	Nucleotide and amino acid moderate conserved. Moderate physicochemical difference between Pro and Leu (Grantham dist.: 98 [0‐215]), Align GVGD: C0 (GV: 138.04 ‐ GD: 16.18), SIFT: Tolerated (score: 0.25, median: 3.33), MutationTaster: disease causing (*P*‐value: 1), PolyPhen: BENIGN with a score of 0.434
POLD2Exon 2	NM_001127218.1	c.203G>T p.Arg68Leu	*n* = 1 (#18 = HNPCC)	No rs number	No frequency data	Nucleotide and amino acid moderately conserved. Moderate physicochemical difference between Arg and Leu (Grantham dist.: 102, Align GVGD: C0 (GV: 165.62 ‐ GD: 0.00), SIFT: Tolerated (score: 0.39, median: 2.97), MutationTaster: disease causing (*P*‐value: 1),PolyPhen: BENIGN with a score of 0.195
RPA1Exon 12	NM_002945.3	c.1160G>A p.Gly387Asp	*n* = 1 (#28 = EC)	No rs number	No frequency data	Nucleotide and amino acid highly conserved. Moderate physicochemical difference between Gly and Asp (Grantham dist.: 94 [0–215]), Align GVGD: C0 (GV: 206.04 ‐ GD: 82.83), SIFT: Deleterious (score: 0, median: 2.86), MutationTaster: disease causing (*P*‐value: 1), PolyPhen: PROBABLY DAMAGING with a score of 0.998
RPA2Exon 8	NM_002946.3	c.731A>G p.Gln244Arg	*n* = 1 (#25 = EC)	No rs number	No frequency data	Moderately conserved nucleotide and amino acid. Small physicochemical difference between Gln and Arg (Grantham dist.: 43 [0‐215]), Align GVGD: C0 (GV: 142.23 ‐ GD: 0.00), SIFT: Tolerated (score: 0.64, median: 2.84), MutationTaster: disease causing (*P*‐value: 1), PolyPhen2: BENIGN with a score of 0.015

**Table 5:** Polymorphisms or probably benign variants detected in the sample cohort (variants of unknown clinical significance)

GeneExon	Ref. sequence	Variant	Found in no. of cases (sample ID)	Rs number	MAF	Comments
EXO1Exon 5	NM_003686.4	c.493C>A p.Gln165Lys	*n* = 20 (#1, 2, 3, 6, 8, 9 10, 11/31, 12, 18, 20, 22, 23, 24, 25, 26, 27, 28, 32, 35)	rs78172944	No frequency data	
EXO1Exon 7	NM_003686.4	c.836A>G p.Asn279Ser	*n* = 1 (#24 = EC)	rs4149909	0.019	
EXO1Exon 9	NM_003686.4	c.1061A>G: p.His354Arg	*n* = 22^a^ (#1, 6, 8, 9, 12, 13, 14, 17, 18, 19, 20, 21, 22, 23, 24, 26, 27, 28, 29, 30, 33, 35)	rs735943	0.348	
EXO1Exon 10	NM_003686.4	c.1316C>T p.Thr439Met	*n* = 5 (#3, 6, 13, 21, 33)	rs4149963	0.090	
EXO1Exon 10	NM_003686.4	c.1372G>A p.Val458Met	*n* = 10 (#1, 13, 21, 22, 23, 24, 26, 27, 28, 35)	rs4149965	0.118	
EXO1Exon 11	NM_003686.4	c.1765G>A p.Glu589Lys	*n* = 18^a^ (#1, 2, 3, 8, 10, 12, 13, 17, 18, 19, 20, 21, 25, 26, 28, 29, 32, 33)	rs1047840	0.361	
EXO1Exon 11	NM_003686.4	c.2009A>G p.Glu670Gly	*n* = 24^a^ (#1, 2, 3, 6, 8, 9, 10, 11/31, 12, 13, 14, 17, 19, 21, 22, 23, 24, 26, 27, 28, 29, 32, 33, 35)	rs1776148	0.295	
EXO1Exon 12	NM_003686.4	c.2167C>T p.Arg723Cys	*n* = 28^a^ (#1, 2, 3, 6, 8, 9, 10, 11/31, 12, 13, 14, 17, 18, 19, 20, 21, 22, 23, 24, 25, 26, 27, 28, 29, 30, 32, 33, 35)	rs1635498	0.084	
EXO1Exon 13	NM_003686.4	c.2270C>T p.Pro757Leu	*n* = 11^1^ (#3, 6, 9, 12, 13, 14, 17, 23, 29, 33, 35)	rs9350	0.260	
LIG1Exon 22	NM_001289064.1	c.2053G>A: p.Val685Met	*n* = 2 (#11/31, 22)	rs146309259	<0.01	
MLH1Exon 8	NM_000249.2	c.655A>G p.Ile219Val	*n* = 13^a^ (#1, 8, 10, 12, 17, 18, 19, 21, 23, 27, 29, 32, 35)	rs1799977	0.172	LOVD MLH1_00294; class 1 Not pathogenic
MLH1Exon 18	NM_000249.2	c.2101C>A p.Gln701Lys	*n* = 1 (#6 = HNPCC)	rs63750114	0.002	LOVD: MLH1_00751, class 2 Likely not pathogenic
MLH3Exon 2	NM_001040108.1	c.691A>C p.Lys231Gln	*n* = 1 (#21 = EC)	rs28756981	0.007	Weakly conserved nucleotide (phyloP: 1.58 [‐14.1;6.4]), Moderately conserved amino acid, Small physicochemical difference between Lys and Gln (Grantham dist.: 53 [0‐215]), Align GVGD: C0 (GV: 146.68 ‐ GD: 0.00), SIFT: Tolerated (score: 0.2, median: 3.28), MutationTaster: polymorphism (*P*‐value: 0.999)
MLH3Exon 2	NM_001040108.1	c.1258G>A p.Val420Ile	n = 1 (#10 = HNPCC)	rs28756982	0.006	Weakly conserved nucleotide (phyloP: 0.85 [‐14.1;6.4]), Weakly conserved amino acid, Small physicochemical difference between Val and Ile (Grantham dist.: 29 [0‐215]), Align GVGD: C0 (GV: 353.86 ‐ GD: 0.00), SIFT: Tolerated (score: 0.65, median: 3.44), MutationTaster: polymorphism (*P*‐value: 1)
MLH3Exon 2	NM_001040108.1	c.2476A>G p.Asn826Asp	*n* = 28^a^ (#1, 2, 3, 6, 8, 9, 10, 11/31, 12, 13, 14, 17, 18, 19, 20, 21, 22, 23, 24, 25, 26, 27, 28, 29, 30, 32, 33, 35)	rs175081	0.007	
MLH3Exon 2	NM_001040108.1	c.2531C>T p.Pro844Leu	*n* = 23 (#1, 2, 3, 8, 9, 10, 11/31, 12, 14, 18, 19, 20, 21, 22, 23, 25, 26, 27, 29, 30, 32, 33, 35)	rs175080	0.359	
MLH3Exon 2	NM_001040108.1	c.2896T>C p.Ser966Pro	n = 2 (#11/31, 13)	rs17782839	0.009	
MSH2Exon 6	NM_000251.2	c.965G>A p.Gly322Asp	n = 1 (#11/31)	rs4987188	0.009	LOVD: MSH2_00275, class 1 Not pathogenic
MSH3Exon 1	NM_002439.4	c.178_186del p.Ala60_Ala62del	*n* = 10 (#2, 3, 12, 13, 20, 26, 28, 30, 32, 33)	No rs number		In‐frame deletion
MSH3Exon 1	NM_002439.4	c.169_195del p.Ala57_Ala65del	*n* = 11 (#2, 3, 12, 13, 19, 20, 26, 28, 30, 32, 33)	No rs number		In‐frame deletion
MSH3Exon 1	NM_002439.4	c.199_207del p.Pro67_Pro69del	*n* = 11 (#2, 3, 12, 13, 19, 20, 26, 28, 30, 32, 33)	rs144629981		In‐frame deletion
MSH3Exon 1	NM_002439.4	c.235A>G p.Ile79Val	*n* = 26^a^ (#1, 2, 3, 6, 9, 10, 11/31, 12, 13, 17, 18, 19, 20, 21, 22, 23, 24, 25, 26, 27, 28, 29, 30, 32, 33, 35)	rs1650697	0.227	
MSH3Exon 21	NM_002439.4	c.2846A>G p.Gln949Arg	*n* = 28^a^ (#1, 2, 3, 6, 8, 9, 10, 11/31, 12, 13, 14, 17, 18, 19, 20, 21, 22, 23, 24, 25, 26, 27, 28, 29, 30, 32, 33, 35)	rs184967	0.107	
MSH3Exon 23	NM_002439.4	c.3133G>A p.Ala1045Thr	*n* = 28^a^ (#1, 2, 3, 6, 8, 9, 10, 11/31, 12, 13, 14, 17, 18, 19, 20, 21, 22, 23, 24, 25, 26, 27, 28, 29, 30, 32, 33, 35)	rs26279	0.312	
MSH3Exon 23	NM_002439.4	c.3232G>A: p.Val1078Ile	*n* = 1 (#18 = HNPCC)	No rs number	no frequency data	Weakly conserved nucleotide, moderately conserved amino acid, Small physicochemical difference between Val and Ile (Grantham dist.: 29 [0‐215]), Align GVGD: C0 (GV: 353.86 ‐ GD: 0.00), SIFT: Tolerated (score: 0.5, median: 3.46), MutationTaster: polymorphism (p‐value: 0.998), PolyPhen: BENIGN with a score of 0.239
MSH6Exon 1	NM_000179.2	c.116G>A p.Gly39Glu	*n* = 10 (#18, 19, 20, 22, 23, 25, 26, 28, 30, 32)	rs1042821	0.193	LOVD: MSH6_00010, class 1 Not pathogenic
MSH6Exon 2	NM_000179.2	c.431G>T p.Ser144Ile	*n* = 1 (#11/31)	rs3211299	0.002	LOVD: MSH6_00028, class 1 Not pathogenic
PMS1Exon 4	NM_000534.4	c.374G>A p.Gly125Asp	*n* = 1 (#19 = EC)	No rs number	No frequency data	Nucleotide weakly conserved and amino acid moderate conserved. Moderate physicochemical difference between Gly and Asp (Grantham dist.: 94 Align GVGD: C0 (GV: 244.82 ‐ GD: 61.50),SIFT: Tolerated (score: 0.21, median: 3.35), MutationTaster: polymorphism (p‐value: 0.848), PolyPhen: BENIGN with a score of 0.271
PMS1Exon 5	NM_000534.4	c.605G>A p.Arg202Lys	*n* = 1 (#33 = HNPCC)	rs2066459	0.009	
PMS2Exon 2	NM_000535.5	c.59G>A p.Arg20Gln	*n* = 2 (#6, 23)	rs10254120	0.07	LOVD: PMS2_00008, class 1 Not pathogenic
PMS2Exon 11	NM_000535.5	c.1408C>T p.Pro470Ser	*n* = 15^a^ (#1, 2, 6, 10, 14, 17, 18, 20, 22, 24, 26, 27, 29, 30, 35)	rs1805321	0.394	LOVD: PMS2_00116, class 1 Not pathogenic
PMS2Exon 11	NM_000535.5	c.1454C>A p.Thr485Lys	*n* = 4 (#8, 9, 14, 19)	rs1805323	0.113	LOVD: PMS2_00138, class 1 Not pathogenic
PMS2Exon 11	NM_000535.5	c.1531A>G p.Thr511Ala	*n* = 2 (#3, 11/31)	rs2228007	0.012	LOVD: PMS2_00071, class 1 Not pathogenic
PMS2Exon 11	NM_000535.5	c.1621G>A p.Glu541Lys	*n* = 25^a^ (#1, 2, 3, 6, 8, 9, 10, 11/31, 12, 13, 14, 17, 18, 19, 20, 22, 23, 24, 25, 26, 27, 28, 30, 33, 35)	rs2228006	0.121	LOVD: PMS2_00028, class 1 Not pathogenic
PMS2Exon 11	NM_000535.5	c.1789A>T p.Thr597Ser	*n* = 2 (#18, 30)	rs1805318	0.005	LOVD: PMS2_00145, class 1 Not pathogenic
POLD1Exon 2	NM_001256849.1	c.56G>A p.Arg19His	*n* = 1 (#6 = HNPCC)	rs3218773	0.009	Weakly conserved nucleotide and moderate conserved amino acid.
POLD1 Exon 2	NM_001256849.1	c.88C>T p.Arg30Trp	n = 1 (#18 = HNPCC)	rs3218772	0.005	Weakly conserved nucleotide and amino acid.
POLD1Exon 4	NM_001256849.1	c.356G>A p.Arg119His	*n* = 2 (#18, 27)	rs1726801	0.162	
POLD1Exon 20	NM_001256849.1	c.2546G>A p.Arg849His	*n* = 2 (#11/31)	rs3218775	0.006	Weakly conserved nucleotide and amino acid.
RPA1Exon 11	NM_002945.3	c.1051A>G p.Thr351Ala	*n* = 4 (#10, 11/31, 16, 30)	rs50330755	0.043	

a Includes the LS sample used as a positive control.

### Exonic variants and clinical interpretation

We identified five exonic insertion/deletion (indel) variants, which included the pathogenic class 5 *MSH2* c.187_188insGG (LS #17) variant previously identified with Sanger sequencing in the LS sample used as a positive control (see Table 3 for details). In addition, a class 5 *MSH6* exon 3 deletion (c.458_627del, EC #20) identified by Pindel, was detected in one EC case which we validated using MLPA (see Table 3 for details). This frameshift deletion was identified in a patient that was diagnosed with EC at the age of 60 years, who also had a diagnosis of CRC but no family history of disease. Both variants, *MSH2* c.187_188insGG and *MSH6* c.458_627del, are frameshift mutations disrupting the normal reading frame and the cause of cancer development in these patients.

Three *MSH3* nonframeshift deletions were also detected in exon 1 using GATK in up to 11 cases (both HNPCC and EC patients, see Table 5 for details). One of the deletions had been reported previously and is listed in the SNP database used by ANNOVAR. All three variants are classified as polymorphisms or probably benign variants as they are not disrupting the reading frame. Ten patients had all three variants, while one patient had two of the three variants.

### Nonsynonymous single‐nucleotide variants (SNVs) and clinical interpretation

We detected 42 nonsynonymous SNVs in thirteen different genes (listed in Tables 3, 4, and 5 according to variant classification). One class 4 SNV was predicted to affect splicing, *MLH1* c.116G>A (EC #21) was found to be likely pathogenic (Table 3) and was validated using Sanger sequencing. This variant was identified in one EC patient who was diagnosed at the age of 57 years, who also had a diagnosis of bowel polyps. The patient has two sisters with both EC and CRC diagnosis.

Four novel SNVs; *LIG1* c.980C>T (EC #24), *POLD2* c.203G>T (HNPCC #18), *RPA1* c.1160G>A (EC #28) and *RPA2* c.731A>G (EC #25) were classified as variants of uncertain significance (class 3) and are listed in Table 4. All four SNVs were found in only one sample and had at least one annotation score indicating that the variant is deleterious or disease causing.

In Table 5 we have listed the variants classified as polymorphisms or probably benign variants; 37 of these are SNVs. Twelve of these were detected in a number of samples, including the LS case used as a positive control. Nine of the SNVs were seen in only one sample. The minor allele frequency (MAF, from 1000 genomes project) in these nine SNVs is very low or no frequency data is reported.

### Intronic variants and clinical interpretation

The average number of intronic variants identified was ~2000–2500 per sample across the 22 genes. One of these, an intronic variant in *EXO1*, was identified by ANNOVAR to affect splicing and the consequence of this change is not predictable, but a skip of exon 15 is very likely (see Table 4). The *EXO1* c.2212‐1G>C (HNPCC #18) was identified in one HNPCC patient diagnosed with CRC at the age of 68 years. IHC from the patients tumor showed lack of MLH1 expression and a common polymorphisms in *MLH1* was identified in this patient with Sanger sequencing during mutation screening (*MLH1* c.655A>G, p.Ile219Val – classified as class 1 in LOVD). The patient has a family history of CRC, uterine cancer, and melanoma.

### Internal control sample (replicate sample)

**Figure 1:**
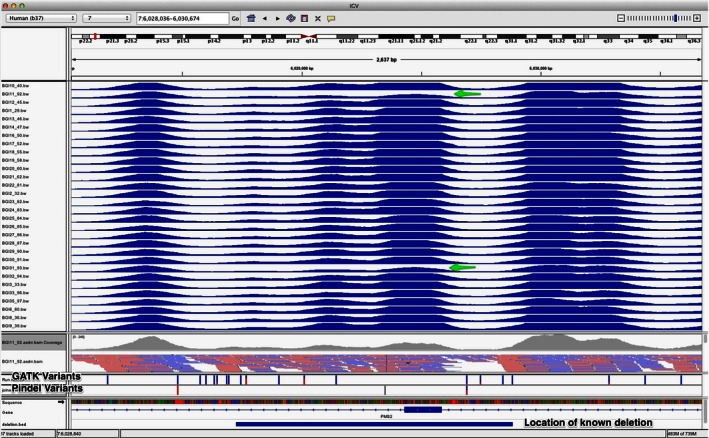
Integrative Genomics Viewer (Broad Institute) was used to plot the coverage of all individuals in the region of the *PMS2* gene covering exon 10. The technology (NimbleGen human custom array) shows a dip in coverage in this genomic region for sample #11 and 31, which may be indicative of a heterozygous deletion.

We chose one sample to act as an internal control sample (replicated – sample #11 and #31). As seen in Table 1 the patient carries two common polymorphisms in *MSH2* and *MSH6* in addition to an exon deletion in *PMS2,* all previously detected with Sanger sequencing. The two common polymorphisms were detected in both samples and 95.99% of the genotypes (variants and reference‐conform calls) between the two samples are the identical. The *PMS2* exon deletion was not detected with the computational tools used in this study. But after further investigation, the technology (NimbleGen human custom array) shows a dip in coverage in this genomic region for sample #11 and 31, which may be indicative of a heterozygous deletion (see Fig. 1).

## Discussion

From the 26 HNPCC and EC patients screened with targeted NGS, we have identified two exonic variants that are consistent with a diagnosis of LS in two EC patients (one class 5; *MSH6* c.458_627del and one class 4 variant; *MLH1* c.116G>A). The *MSH6* exon 3 deletion has previously been reported and is predicted to change the function of the gene 33. The SNV in *MLH1* is considered to affect splicing and has also previously been reported 34, and is listed multiple times in Leiden Open Variation Database (LOVD). The patients carrying the *MSH6* and *MLH1* variants had EC diagnosed years later than the average age of cancer development in LS patients (60 and 57 years of age, respectively) and neither of the two were carriers of any additional variants that was considered to be of significance. There was no family history of cancer reported in the patient harboring the *MSH6* deletion, while the patients carrying the *MLH1* variant has a sister diagnosed with EC and CRC. Both patients had high BMI (see Table 2) placing them in the obese category, which is atypical in LS.

In one HNPCC patient sample (#18, CRC at 68 years of age) that showed an absence of *MLH1* expression by IHC, an intronic variant in *EXO1* (intron 14) was detected which was predicted to affect splicing. The same variant was first reported by Wu et al. 35. It was dismissed as a mutation as it was later detected in three Dutch controls (3/704) 36 and therefore, it seems unlikely to represent a disease‐causing mutation. In this context, it should be noted that there are two enzymatically active alternate splice forms of *EXO1*, one of which contains exon 14 and the other is truncated after exon 13 37. The two forms are a result from alternative RNA splicing and the predicted proteins differ at a small region of the COOH terminus of each protein 37. Interestingly, the region of *EXO1* that interacts with *MSH2* is located in the COOH terminus 38. Human *EXO1* is a 5′ to 3′ exonuclease that directly interacts with *MSH2*,* MSH3* and *MLH1,* and is thought to stabilize higher order complexes of MMR proteins 39, 40, 41.. The gene participates in DNA MMR and possibly the DNA recombination function of *MLH1* 39. This could explain the observed absence of MLH1 expression assessed by IHC in this patient. But methylation of promoter region of *MLH1* cannot be ruled out as an explanation for the absence of MLH1 expression 42 even though this is most likely not the case for this family that has reported a strong history of CRC (methylation of *MLH1* is usually seen in sporadic CRC cases). Mutations in *EXO1* have also been associated with late onset CRC or atypical HNPCC and a weak mutator phenotype but when combined with additional weak mutator alleles can increase genetic instability 43, as observed in the patient in the current study.

*EXO1* appears to act as a modifier genes rather than a highly penetrant germline mutation 35. The results from this study supports this hypothesis as the patient harboring the *EXO1* intronic variant also harbored additional variants, not observed in any of our other patients, that included one class 3 variants (*POLD2,* listed in Table 4) and two class 1 or 2 variants (*MSH3* and *POLD1,* listed in Table 5). The patient developed CRC at the age of 68 and had a strong family history of CRC with later age onset, supporting the notion that changes in *EXO1* are not associated with younger ages of disease onset. These findings suggest that the patient may be harboring multiple low penetrance variants that potentially increase cancer risk at later ages of onset. Only one other patient (HNPCC #6) harbored multiple variants; two class 1/2 variants (*MLH1* c.2101C>A and *POLD1* c.56G>A). Interestingly, this patient also shows a lack of *MLH1* expression as judged by IHC.

Three novel class 3 variants, *LIG1* c.980C>T, *RPA1* c.1160G>A and *RPA2* c.731A>G, were identified in individual EC patients that were not observed in any other subjects. These genes play multiple roles in human MMR and the variants will require further investigation to determine if they play a role in disease development. Four other patients each carried an SNV classified as class 1 or 2, two in *MLH3* and two in *PMS1*. Both *MLH3* variants were deemed to be tolerated and were classified as common polymorphisms with reported minor allele frequencies (MAF) of 0.006 and 0.007. Similarly, one of the *PMS1* variants was tolerated and considred benign with a MAF = 0.009, whereas the second *PMS1* variant (c.605G>A) had previously been reported to have a functional significance via the alteration of exonic splicing enhancers as judged by in silico analysis 44.

The present study has some potential limitations. We acknowledge that NGS technology development is so rapid that library preparation and number of total effective reads in this study do not conform to current standards. However, as we detected the previously identified missense variants in our replicates and genotype accuracy is almost 96% (which is over what is expected from this technology^45^ we argue that our approach is appropriate for the presented results. Pseudogenes of *PMS2* present challenges but can be overcome with proper primer design 46, or elimination of exon 12 and 15 during analysis 47. We did not specifically optimise the primer design for this study and the coverage of *PMS2* was the lowest of all genes. Even though the NimbleGen tiled region has probes covering exon 10, the *PMS2* exon 10 deletion (in sample #11/31) was not detected by the computational tools used in this study. While GATK does not call large deletions, we speculate that PinDel did no call this deletion due to the uneven read coverage in this region. The coverage fluctuation is due to *PMS2* having many pseudogenes and one of them; *PMS2CL*, lacks exon 10. This inflates the coverage across the whole gene except for exon 10 where no additional reads are contributed from the pseudogene. Therefore, the additional coverage drop in sample #11/31 is not detected as a deletion as there is already a general coverage drop across all samples at this location. However, visually inspecting the coverage across all samples at this location indicates the expected deletion to be present, see Figure 1. The sample size is small and the samples have been screened for mutations in *MLH1*,* MSH2*,* MSH6* and/or *PMS2* previously and can explain why we have not detected any class 4/5 variants in any of the HNPCC cases included in the study. Another possible limitation is the fact that tumor tissue was not available for IHC/MSI for all the samples included in the study.

In the analysis presented here we have focused on exonic variants and nonsynonymous SNVs. We have not addressed the presence of absence of all intronic variants, even though there is an enormous amount of data to be investigated. SNVs that are not in protein‐coding regions (synonymous SNVs) was not the focus of this study, but may still affect messenger RNA splicing, stability and structure, transcription factor binding as well as protein folding, which can have significant effect on the function of the protein 48.

In conclusion, by utilising new technology we have identified two LS families from the EC pateints, one without a family history of cancer and high BMI (obese), supporting the notion of universal screening of all EC patients. In addition, we have detected three novel class 3 variants to be followed up in EC cases. This study provides evidence that MMR screening provides new information about genetic risk for patients diagnosed with HNPCC and that genes not routinely tested for can play a role in cancer development in HNPCC patients through polygenic interactions that may indeed be causative. More patients need to be tested but there is the potential for rapid uptake of new testing strategies to improve risk assessment and prophylactic measures to redudce the burden of disease in this susceptible group of patients. Finally, the discovery of new genetic loci affecting the risk of developing cancer in this population will also have implications for cancer patients in the general population as the polygenic interaction reported herein may confer novel insight into new pathways for cancer development.

## Conflict of Interest

None declared.
